# Comparative analysis of co-culture and monoculture models in simulating diabetic neurovascular dysfunction: insights into diabetic retinopathy

**DOI:** 10.3389/fendo.2023.1215218

**Published:** 2023-09-08

**Authors:** Qiyun Wang, Zhixin Qiao, Wenting Kang, Ling Zhu, Xinyuan Zhang

**Affiliations:** ^1^Beijing Tongren Eye Center, Tongren Hospital, Capital Medical University, Beijing, China; ^2^Beijing Retinal and Choroidal Vascular Disorders Study Group, Beijing, China; ^3^Clinical Research Center, Tongren Hospital, Capital Medical University, Beijing, China; ^4^Save Sight Institute, Department of Ophthalmology, Faculty of Medicine and Health, The University of Sydney, Sydney, NSW, Australia

**Keywords:** diabetic neurovascular dysfunction, retinal vascular endothelium cells, ganglion cells, co-culture, monoculture

## Abstract

**Background:**

Interaction between retinal vascular endothelial cells and neurons plays a critical role in the pathogenesis of diabetic retinopathy (DR). This study aims to compare an *in vitro* model over a monoculture model to simulate the neurovascular coupling under the hyperglycemic microenvironment of diabetes.

**Methods:**

Rat retinal vascular endothelial cells (RRMECs) and ganglion cells (RGCs) were seeded mono- or co-cultured in a normal (NG, 5.5 mM) and high (HG, 75 mM) glucose concentrations culture medium. Cell viability was detected by the cell counting kit-8 (CCK-8) assay. The ability of migration and lumen formation of RRMECs were determined by scratch wound, transwell migration, and lumen formation assays. The apoptosis index of cells was calculated and detected by propidium iodide (PI)/Hoechst staining. Quantitative and morphological analysis of RGCs was performed through the labeling of RGCs by brain-specific homeobox/POU domain protein 3A (BRN3A) and anti-beta-III tubulin (TUJ1). The gene and protein expression levels of occludin (OCLN) and zonula occludens-1 (ZO-1) were evaluated by quantitative real-time polymerase chain reaction and enzyme-linked immunosorbent assay.

**Results:**

The viability, migration, and lumen formation abilities of RRMECs in the HG group significantly increased (*P*<0.05) in both mono- and co-culture models. Migration and lumen formation abilities of RRMECs in the co-culture with HG were lower than that in the monoculture group (*P*<0.05). The viability of RGCs cells with HG significantly decreased in both mono- and co-culture models (*P*_mono_<0.001, *P*_co_<0.001), the apoptosis index of RGCs in the co-culture with HG was higher than that in the monoculture (*P*=0.010). The protein and gene expression of OCLN, and ZO-1 in RRMECs significantly decreased with HG culture medium in both culture models (*P*<0.05). In the HG group, the protein and gene expression level of the ZO-1 and OCLN of RRMECs significantly decreased in the co-culture model than that in the monoculture model (*P*<0.05).

**Conclusion:**

Compared with mono cell culture, the established co-culture *in vitro* system for diabetic neurovascular dysfunction can better stimulate the micro-environment of the retinal neurovascular unit.

## 1 Introduction

Diabetic retinopathy (DR) is a neurovascular disorder (1). Retinal neurovascular unit (RNVU), which describes the intricate functional coupling and interdependency among neurons, glial cells, and blood vessels, was introduced after first unveiling the concept of the brain neurovascular unit in the central neuronal system. Numerous studies have shown that RNVU dysfunction is a critical characteristic of DR (2–4). Among the RNVU, microvascular endothelial cells have been found to regulate differentiation, guide migration, and promote the survival of neurons (5). Furthermore, neurons regulate blood vessel size and blood flow by supporting cells in the microenvironment (such as glial cells) in response to retinal activity through a mechanism known as neurovascular coupling. In recent years, the interaction between retinal neurons and vascular endothelial cells has been a new direction for studying the pathogenesis of DR.

The cell culture technique, which was developed by Ross Harrison in the first decade of the twentieth century to investigate animal cell behavior *in vitro*, has provided numerous pivotal information for understanding the pathogenesis of various diseases, including DR (6). Our previous study successfully established a mono cell culture DR *in vitro* model. We found that glucose of 25 mM to 50 mM was the appropriate range and 100 mM was the extreme value of hyperglycemia for human retinal microvascular endothelial cells (HRMECs) *in vitro*; 50 mM to 150 mM was the proper range for rat retinal ganglion cells (RGCs) (7). In this study, we aim to establish a neuron and endothelial co-culture model to simulate the environment of neurovascular coupling. We further test the hypothesis that the co-culture system can better stimulate the neurovascular coupling in RNVU under hyperglycemia.

## 2 Materials and methods

### 2.1 Chemicals and reagents

Dulbecco’s modified Eagle’s medium (DMEM), fetal bovine serum (FBS), 0.25% trypsin, Hank’s Balanced Salt Solution, and phosphate buffer saline (PBS) were bought from Gibco Life Technologies (New York, USA). Cell counting kit-8 (CCK8), propidium iodide (PI), 4’,6-diamidino-2-phenylindole (DAPI), and Hoechst were purchased from Sigma-Aldrich (St. Louis, USA). 6-well cell culture plate, 24-well cell culture plate, transwell plates with 0.4μm and 0.8μm pore size of microporous membrane, and T25 cell culture bottle were purchased from Costar (Corning, USA). Matrigel (Cat#356234) was purchased from BD Biosciences (Oxford, UK). Other chemicals, including rabbit anti-beta-III tubulin (TUJ1) antibody (Cat#ab18207), goat anti-rabbit (Cat# Cat#ab150116), and goat anti-mouse FITC fluorescent (Cat#ab150077) antibody (Abcam, Cambridge, Britain), mouse anti-brain-specific homeobox/POU domain protein 3A (Brn3a) (Cat#sc-8429, Santa, Cruz, USA), occludin (OCLN), zonula occludens-1 (ZO-1) ELISA kit (Cloud-Clone Crop, Wuhan, China), Trizol solution (Biomiga, San Diego, USA) and cDNA synthesis kit (SYBR qPCR Master Mix, Novoprotein, Shanghai, China) were purchased previously.

### 2.2 Co-culture and monoculture

Commercially available rat retinal microvascular endothelial cells (RRMECs) and RGCs (Xuanya Biotechnology Co. LTD, Shanghai, China) were cultured in DMEM supplemented with 10% FBS. RRMECs and RGCs were grown in a humidified atmosphere containing 5% CO^2^ at 37°C. When the two types of cells grew to about 90% fusion and showed monolayer adherent growth like paving stones, 0.25% trypsin was added for digestion and moved to cell plate 1:3 passage culture. Cells from passages 4–6 in culture were used in this study.

**Figure 1:**
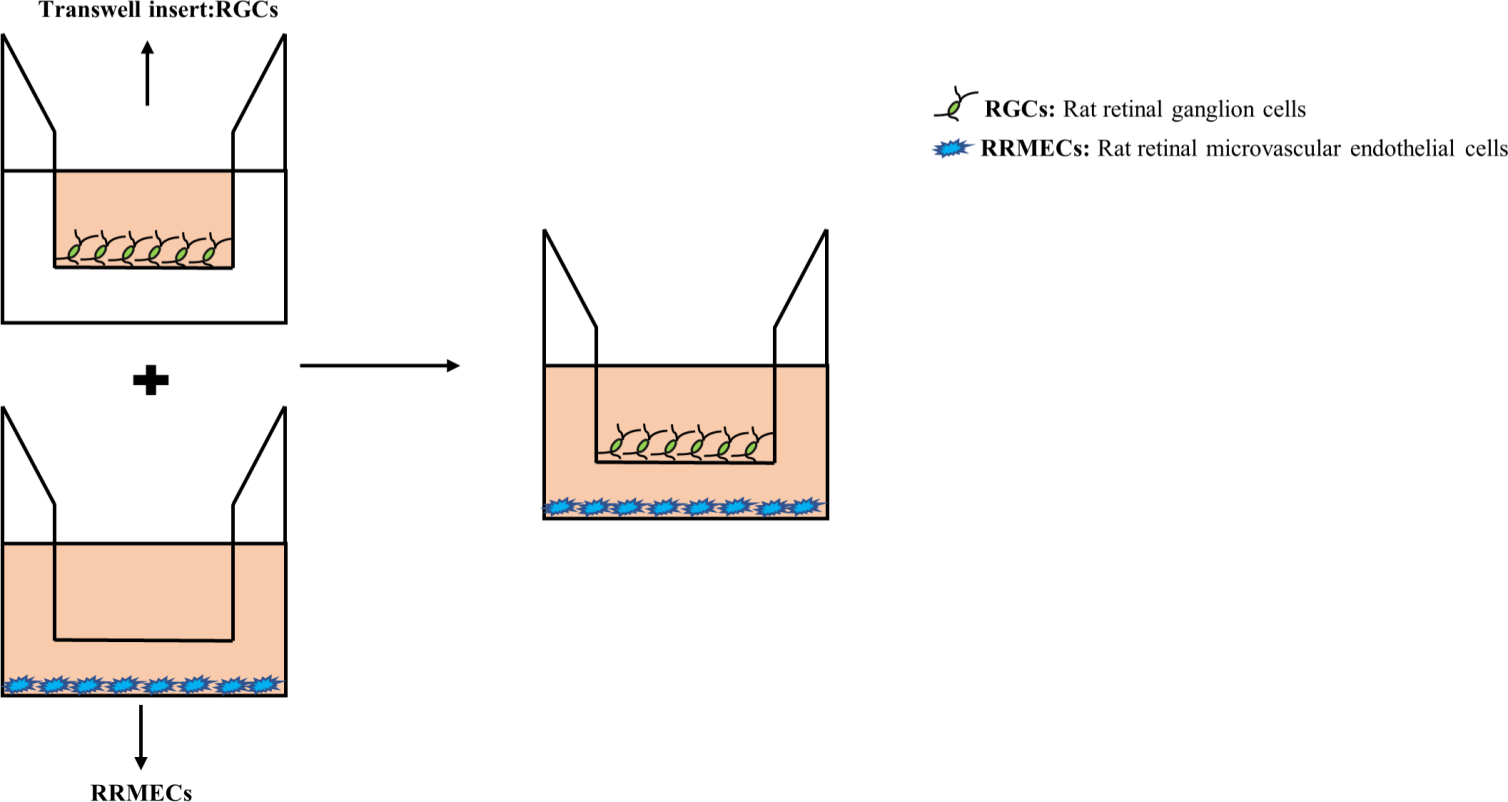
An RRMECs and RGCs *in vitro* co-culture model for hyperglycemia. After the upper and lower chambers were seeded with RGCs and RRMECs respectively, and after the cells adhered, transwell chambers (0.4μm and 8μm in pore size) were inserted into a 24-well plate. RRMECs, Rat retinal microvascular endothelial cells; RGCs, Rat retinal ganglion cells.

Transwell co-culture system (0.4μm) was assembled by using 2×10^4^ RGCs and 6×10^4^ RRMECs (at a ratio of 1:3) as we described previously (8, 9). RRMECs were seeded in 24-well plates, and RGCs were planted in the transwell. After 24h, transwells containing RGCs were moved into the 24-well plates containing RRMECs to establish a co-culture system (**Figure 1**).

The cell culture medium of the control group contained normal glucose DMEM medium (5.5mmol/L glucose, NG) with 10%FBS. As we described previously (7), the culture media containing 75mmol/L glucose was the high glucose group (HG). The subgroups of this experiment mainly included: a monoculture group with NG, a monoculture group with HG, a co-culture group with NG, and a co-culture group with HG. In this experiment, five duplicated wells were set in each group, 48h time point of culture duration was selected according to our previously described.

### 2.3 Cell viability measurement by CCK-8

The concentration of CCK-8 solution was adjusted to 10% of the total volume of PBS in the upper and lower chambers. After gently mixing, cells were incubated for 2 hours in the dark. The OD value of each well was detected at the wavelength of 450nm using a microplate reader (Multiskan MK3, Thermo, CA, USA).

### 2.4 Cell migration ability measurement using scratch wound and transwell assays

#### 2.4.1 Scratch wound assay

When RRMECs cells in the lower compartment grew to more than 90%, the monolayer was scratched using a tip and washed with PBS to remove detached cells. At 0h and 48h after the scratch, three visual fields were photographed under a microscope (Carl Zeiss, Jena, Germany) and the scratch area was measured by Image-J analysis software (National Institutes of Health, USA). The closure area of the wound was calculated as follows: the cell migration rate (%) = [(scratch area at 0h -scratch area at 48h)/scratch area at 0h] ×100%.

#### 2.4.2 Transwell assay

100ul of RRMECs cell suspension was added into the transwell chamber with a pore size of 8μm, and cell density was adjusted to 2×10^4^/well. 600μl of RGCs cell suspension was added into the 24-well plates with a cell density of 6×10^4^/well. After incubation for 48h, cells were fixed with methanol for 15 minutes and then stained with 0.1% crystal violet for 20 minutes. The cells were observed and counted in 3 fields under a microscope.

### 2.5 Cell apoptosis and quantitative analysis

In mono- and co-culture models, the medium was changed in the upper and lower chambers simultaneously, and each NG and HG group was set with five multiple wells. After 48h, 10μl PI and 10μl Hoechst were added to each well and incubated for 15 minutes in the incubator. Three fields were randomly taken under a microscope, and the number of stained cells was calculated using Image-J software. Cell apoptosis index (AI) was calculated as PI-stained cells/Hoechst-stained cells.

### 2.6 Lumen formation and quantitative analysis

300μl Matrigel was added to a 24-well plate and incubated for 45 minutes at 37° C in a humidified atmosphere with 5% CO_2,_ as previously described. The NG and HG groups suspensions of RRMECs were inoculated into 24 wells pre-coated with Matrigel colloid. RGCs cells were then placed into transwells in 24-well plates. Cells were incubated at 37°C incubator for 6 hours. A microscope was used to observe the status of tube formation. Three different fields were captured, and ImageJ software calculated the number of lumens.

### 2.7 Identification of cell morphology by immunofluorescence electron microscopy

RGCs were incubated with rabbit anti-TUJ1 and mouse anti-Brn3a antibodies as previously described (10, 11). RGCs were then incubated with secondary antibodies (goat anti-mouse and goat anti-rabbit) and counterstained with DAPI. Three photographic fields were randomly selected under a microscope. The number of cells with neurite lengths equal to or greater than three times the cell body diameter was calculated using ImageJ.

### 2.8 Enzyme-linked immunosorbent assay

ZO-1 and OCLN levels in the supernatants of RRMECs were evaluated using an ELISA assay. The ELISA ST-360 microplate reader (at 450nm) was employed for the measurements, following the manufacturer’s protocol.

### 2.9 Real-time PCR for evaluating ZO-1 and OCLN genes

The total RNA was extracted using a Trizol solution. The cDNA of different samples was synthesized using 2μg of total RNA and the transcription first-strand cDNA synthesis kit. The designed primers for OCLN, ZO-1, and β-actin are shown in **Table 1**. Experimental parameters for PCR were denaturation at 95°C for 10 minutes, annealing at 60°C for 20 sec, and extension at 72°C for 30 sec for 40 cycles. The density of individual lanes was normalized to the density of the PCR-amplified internal control β-actin.

**Table 1:** Sequences of primers used in RT-PCR.

Genes	GenBank Accession Numbers	Forward primer sequence	Reverse primer sequence	PCR product(bp)
*ZO-1*	XM_039105341.1	5’GCCTCTGCAGTTAAGCAT3’	5’AAGAGCTGGCTGTTTTAA3’	249
*OCLN*	XM_039103245.1	5’CTGTCTATGCTCGTCATCG3’	5’CATTCCCGATCTAATGACGC3’	294
*β-actin*	AA_874855	5’TTCCACACACACCAGCTTCG3’	5’GGGGTGGTGTGGAGATTTAG3’	366

ZO-1, Zonula occludens-1; OCLN, Occludin, RT-PCR, Real-time polymerase chain reaction.

### 2.10 Statistical analysis

Each experiment was conducted independently and at least three times for statistical analysis. Data normality was assessed by the Shapiro-Wilk test. Student t-tests were performed to analyze the differences between the two groups. The data are shown as the mean ± standard deviation (SD) and were analyzed by one-way analysis of variance (ANOVA), followed by the least significant difference (LSD) test, using SPSS software (SPSS, Inc. 23.0, Chicago, IL, USA). *P*<0.05 was considered statistically significant.

## 3 Results

### 3.1 Comparison of cell viability between the co-culture and monoculture group

**Figure 2:**
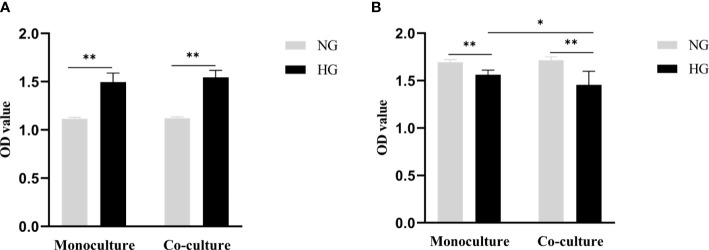
Cell proliferation ability of RRMECs and cell activity of RGCs detected by CCK-8 assay. **(A)** Shows the proliferation ability of RRMECs detected by CCK-8 in HG environment at 48H. **(B)** Shows the cell activity of RGCs detected by CCK-8 in the HG environment at 48H. RRMECs, Rat retinal microvascular endothelial cells; RGCs, Rat retinal ganglion cells; CCK8, Cell counting Kit-8; HG, High glucose; NG, Normal glucose; H, Hours. *The difference is statistically significant, *P*<0.05. **The difference was statistically significant, *P*<0.001.

After 48 hours of RRMECs cultured in the lower compartment pore plate, compared with the NG group, the proliferation ability of cells in the HG group was significantly increased, and the difference between the two groups was statistically significant (1.12 ± 0.01 vs. 1.50 ± 0.09, *P*_mono_<0.001; 1.12 ± 0.02 vs. 1.54 ± 0.07, *P*_co_<0.001) (**Figure 2A**).

The activity of RGCs cells cultured with HG in monoculture and co-culture was significantly inhibited in comparison with the NG group (1.56 ± 0.05 vs. 1.69 ± 0.03, *P*_mono_<0.001; 1.46 ± 0.14 vs.1.72 ± 0.04, *P*_co_<0.001), the difference between the monoculture and co-culture in the HG group was statistically significant (*P*=0.015) (**Figure 2B**).

### 3.2 Comparison of cell migration ability between the co-culture and monoculture group

**Figure 3:**
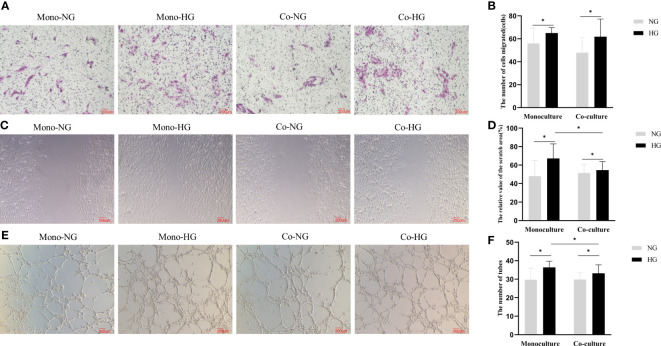
Migration and lumen formation abilities of RRMECs in the monoculture and co-culture models. **(A)** Shows the migration ability of RRMECs detected by transwell at 48H (magnification 200×). **(B)** Shows the number of cells migrated at 48H. **(C)** Shows the migration ability of RRMECs detected by scratch assay at 48H (magnification 200×). **(D)** Shows the relative value of the scratch area. **(E)** Shows the shape of the lumen of each group of cells under the microscope (magnification 200×). **(F)** Shows the comparative analysis of the number of formed cell lumens in each group. RRMECs, Rat retinal microvascular endothelial cells; Co, Co-culture; Mono, Monoculture; HG, High glucose; NG, Normal glucose; H, Hours. *The difference is statistically significant, *P*<0.05.

After being co-cultured for 48 hours, the number of RRMECs in the HG group was significantly higher in comparison with the NG group (62 ± 15 vs. 48 ± 13), and the difference between the two groups in the co-culture model was statistically significant (*P*=0.018). RRMECs migration ability in monoculture with HG was significantly higher in comparison with the NG group (65 ± 5 vs. 56 ± 13, *P*=0.025). The number of migrations in monoculture with HG was higher than that in co-culture without statistically significant (*P*>0.05) (**Figures 3A, B**).

The migration rate of RRMECs treated with HG was higher than the NG group in both monoculture and co-culture models (*P*_mono_=0.003, *P*_co_=0.033). The migration rate in the co-culture model in the HG group was lower than that in the monoculture model (*P*=0.021) (**Figures 3C, D**).

### 3.3 Comparison of the cell lumen formation ability between co-culture and monoculture group

The number of lumens of the monoculture RRMECs in the HG group was higher than that in the NG group (36 ± 3 vs. 30 ± 6, *P*=0.002). The lumen formation ability of RRMECs co-cultured in the HG group was significantly increased in comparison with the NG group (33 ± 4 vs. 30 ± 3, *P*=0.021) than that in the NG group. In the HG group, the lumen formation ability in the monoculture model was more than in the co-culture model (*P*=0.037) (**Figures 3E, F**).

### 3.4 Quantitative analysis of cell apoptosis in the co-culture and monoculture groups

**Figure 4:**
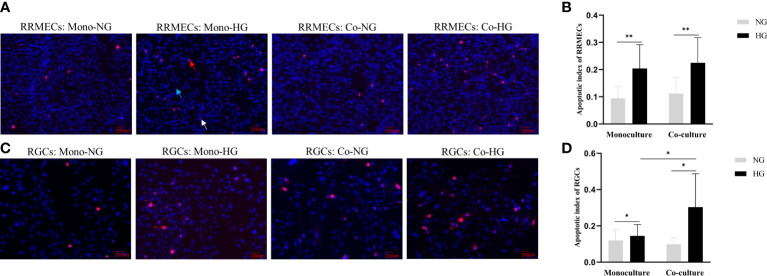
Apoptosis of RRMECs and RGCs in different concentrations of glucose in the monoculture and co-culture models. **(A)** Shows the detection of apoptosis of RRMECs by PI/Hoechst staining (magnification 200×). **(B)** Shows the AI of RRMECs. **(C)** Shows the apoptosis of RGCs detected by PI/Hoechst staining (magnification 200×). **(D)** Shows the AI of RGCs. Blue arrow: Cells were stained by Hoechst; Red arrow: Cells were stained with PI; White arrow: Cells were stained with Hoechst and PI; RGCs, Rat retinal ganglion cells; RRMECs, Rat retinal microvascular endothelial cells; Co, Co-culture; Mono, Monoculture; HG, High glucose; NG, Normal glucose; AI, Apoptotic index. *The difference is statistically significant, *P *< 0.05. **The difference was statistically significant, *P *< 0.001.

In the monoculture model, the AI of RRMECs in the NG and HG groups were (0.09 ± 0.04) and (0.20 ± 0.09), respectively. In the co-culture model, the AI of RRMECs in NG and HG groups were (0.11 ± 0.06) and (0.22 ± 0.09). Compared with the NG group, the AI of RRMECs treated with HG was higher both in the monoculture and co-culture models (*P*<0.001). In the HG group, the AI of RRMECs in the co-culture model was high than in the monoculture model, but there was no statistical difference(*P*>0.05) (**Figures 4A, B**).

PI/Hoechst staining and ImageJ was used to label and quantify the live and apoptotic cells. The AI of RGCs treated with HG in the monoculture and co-culture groups were (0.14 ± 0.06) and (0.30 ± 0.18) respectively, and AI in the NG-treated cells in the monoculture and co-culture groups were (0.12 ± 0.06) and (0.10 ± 0.04). The AI in the HG group was significantly higher than that in the NG group (*P*_mono_=0.004, *P*_co_=0.025). The AI of RGCs in the co-culture model treated with HG was higher than that in the monoculture model (*P*=0.010) (**Figures 4C, D**).

### 3.5 Comparison of the morphological changes of RGCs in the co-culture and monoculture groups

**Figure 5:**
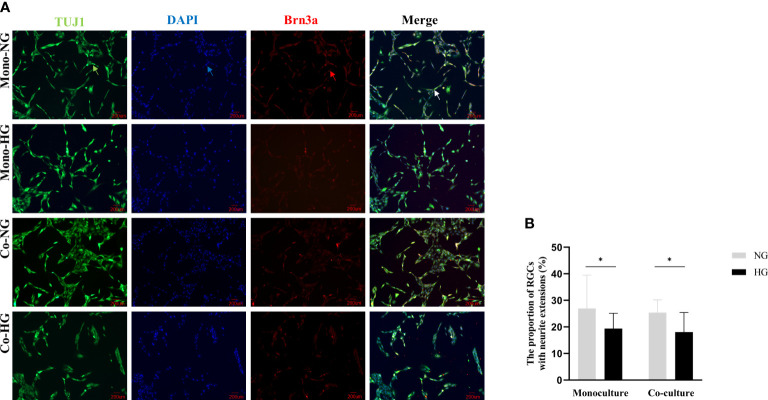
Morphology of RGCs cells in monoculture and co-culture models. **(A)** Shows the morphology of RGCs observed with an inverted fluorescent microscope (magnification 200×). **(B)** Shows the proportion of RGCs with neurite extensions. Green arrow: Rabbit anti-beta-III tubulin-labeled RGCs. Red arrow: Mouse anti-Brn3a-labeled RGCs; Blue arrow: DAPI labeled RGCs nuclei; White arrow: Synthetic plot of cellular immunofluorescence staining of labeled RGCs; RGCs: Rat retinal ganglion cells; Brn3a: Brain-specific homeobox/POU domain protein 3A; DAPI: 4’,6-diamidino-2-phenylindole; Co, Co-culture; Mono, Monoculture; HG, High glucose; NG, Normal glucose. *The difference is statistically significant, *P*<0.05.

RGCs were labeled by neurons and RGCs-specific markers TUJ1 and Brn3a, respectively, followed by the ImageJ quantitative analysis. The proportions of RGCs with neurite extensions in monoculture were (27 ± 13) % in the NG group and (19 ± 6) % in the HG group. The proportions of RGCs with neurite extensions in co-culture were (25 ± 5) % in the NG group and (18 ± 7) % in the HG group. Compared with the NG group, the proportions of RGCs with neurite extensions in the HG group were significantly different (*P*_mono_=0.046, *P*_co_=0.003). There was no statistical difference in the proportions of RGCs with neurite extensions between the two models (*P*>0.05) (**Figure 5**).

### 3.6 Comparison of the protein levels of tight junction proteins in the two culture models

**Figure 6:**
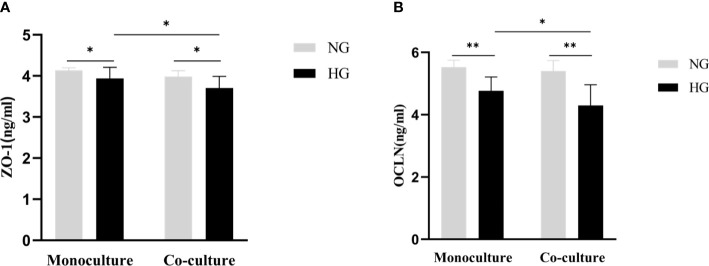
Comparison of the protein expression level of tight junction proteins (ZO-1, OCLN) in the monoculture and co-culture models by ELISA. **(A)** Shows the protein levels of ZO-1 in RRMECs. **(B)** Shows the protein levels of OCLN in RRMECs. RRMECs, Rat retinal microvascular endothelial cells; ZO-1, Zonula occludens-1; OCLN, Occludin; HG, High glucose; NG, Normal glucose. **The difference is statistically significant, *P*<0.001. *The difference is statistically significant, *P*<0.05.

The protein expression level of ZO-1 in HG and NG groups in RRMECs with monoculture was (3.94 ± 0.27 ng/ml vs. 4.14 ± 0.06 ng/ml), and ZO-1 in HG and NG groups with co-culture were (3.71 ± 0.28 ng/ml vs. 3.97 ± 0.14 ng/ml). There was a significantly decreased protein expression level of ZO-1 in the monoculture and co-culture models with HG compared with the NG (*P*_mono_=0.014, *P*_co_=0.003). Similarly, the expression level of OCLN in the two groups with HG was significantly decreased compared to the two groups cultured with NG (*P*_mono_<0.001, *P*_co_<0.001). Compared with the monoculture model, the levels of ZO-1 and OCLN were lower in the co-culture model (*P*_ZO-1 = _0.027, *P*_OCLN_ =0.032) (**Figure 6**).

### 3.7 The expression of the mRNA levels of tight junction proteins in the co-culture and monoculture models

**Figure 7:**
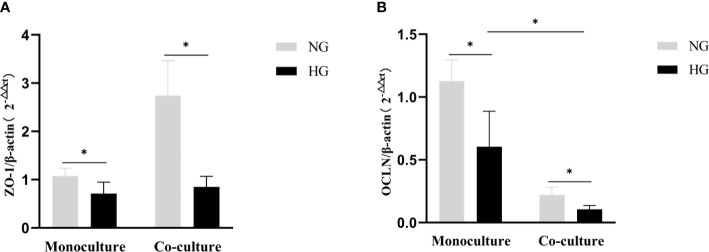
Comparison of gene expression level tight junction proteins (ZO-1, OCLN) by RT-PCR in the monoculture and co-culture models. **(A)** Shows the expression of the mRNA levels of ZO-1 in RRMECs. **(B)** Shows the expression of the mRNA levels of OCLN in RRMECs. RRMECs, Rat retinal microvascular endothelial cells; ZO-1, Zonula occludens-1; OCLN, Occludin; HG, High glucose; NG, Normal glucose. *The difference is statistically significant, *P*<0.05.

The mRNA expression of ZO-1and OCLN decreased in RRMECs monoculture with HG in comparison with the NG group (0.71 ± 0.23 vs. 1.07 ± 0.16, *P*=0.022; 0.60 ± 0.28 vs. 1.13 ± 0.17, *P*=0.008). Similarly, in the co-culture model, the mRNA expression of ZO-1 and OCLN in RRMECs with HG was lower than NG group (0.85 ± 0.22 vs. 2.74 ± 0.73, *P*=0.001; 0.11 ± 0.03 vs. 0.22 ± 0.06, *P*=0.005). Compared to the monoculture model, the mRNA expression of OCLN in the co-culture model treated with HG significantly decreased (*P*=0.004) (**Figure 7**).

## 4 Discussion

This study compared the co-culture and monoculture models to investigate which system can better simulate diabetic neurovascular dysfunction. Migration and lumen formation abilities of RRMECs in the co-culture with HG were lower than that in the monoculture group, due to the lower expression level of tight junction proteins. Similarly, the viability of RGCs cells with HG significantly decreased in both mono- and co-culture models. The apoptosis index of RGCs in the co-culture with HG was higher than in the monoculture. These results indicate that the interactions between the RRMECs and RGCs affect the cell variability morphology of the two cell types. The co-culture model can better stimulate the micro-environment of both endothelial and RGCs under hyperglycemia.

An *in vitro* monoculture of retinal vascular endothelial cells is the common model to study the pathogenesis of various retinal vascular diseases. In our previous study, we set up a rational monoculture system and optimized glucose concentrations to model diabetic retinal endothelial (25-50 mM) or neuronal(50-150 mM) dysfunction to stimulate DR *in vitro*. Although co-culture models have been applied in stimulating the microenvironment in other diseases, however, till now for our best acknowledgment, there have been no reports in DR neurovascular coupling studies. Co-culture models stimulate various pathogenic factors and multiple signaling pathways in microenvironments under different pathological conditions, including hyperglycemia. This study employed a non-contact co-culture method, offering several advantages, including low-cost, high-throughput capabilities, preservation of cell-cell interactions, and superior barrier properties compared to the monolayer model. The co-culture model can also evaluate small molecules (12). Compared to a monoculture of primary cells, this cell line co-culture has the advantages of no contact and better reproducibility by replacing the primary cells with a stable cell line to address the disadvantages of short cell lifespan and individual differences, and for the application of this model in some high throughput analysis. Compared to monoculture, co-culture displays lower sensitivity to toxic reactions while exhibiting heightened sensitivity to inflammatory reactions.

An *in vitro* model is the simplest system to investigate retinal cell pathophysiological changes, particularly the interaction between RNVU cells, providing a potential future management strategy for various retinal diseases, including DR (13). Compared with the monoculture model, the joint participation of cells of the RNVU in response to the stimulus is more suggestive for subsequent studies. For example, the injury of retinal ganglion cells is mediated by retinal Müller cells in DR (14). Neurons regulate local blood flow through glial cells and pericytes to maintain their functions (15–17). The neurovascular coupling effect has also become a hotspot research area in DR. The co-culture model of HRMECs and neurons has been applied to study central nervous system diseases such as cerebral apoplexy (18, 19). In this study, RGCs and RRMECs were co-cultured to investigate the two cell types’ pathophysiological changes and their interdependence and interaction under hyperglycemia. Based on our previous results (7), the defined glucose concentration of the HG group in this study was 75 mmol/L for future neurovascular coupling studies. We found that interactions between RRMECs and RGCs may affect the cell variabilities and can better stimulate the cell micro-environment under hyperglycemia.

The RRMECs and RGCs showed lower cell activity and higher AI by co-culture. In particular, the lumen-forming ability and migration ability of RRMECs decreased, and the number of tubes in the co-culture model was smaller than that in the monoculture model in RRMECs. This may be due to the protein and mRNA expression level of cell tight junction proteins of ZO-1 and OCLN being significantly decreased in the co-culture model than in the monoculture group. ZO-1 and OCLN are essential components of the BRB, playing a critical role in maintaining the normal functions of the BRB (20, 21). The differentially regulated expression level of tight junction proteins indicated that the co-culture model could better stimulate the neuronal and vascular coupling in the micro-environment (19, 22).

Theoretically, the osmotic pressure is one of the influent factors for cell biological activity under the culture conditions of high glucose concentrations. In our previous study, we set up a mannitol hyperosmotic control group to investigate the effect of elevated Osmotic pressure on retinal endothelial cells and retinal ganglion cell activity using CCK8 assay. In the published study, mannitol was added to the 5.5 mmol/L group to adjust the osmotic pressure to correspond with the same osmotic pressure of G25, G50, G100, and G150 (D-glucose concentrations 25, 50, 100, and 150 mmol/L, respectively), establishing M25, M50, M100, and M150 (mannitol concentrations 19.5, 44.5, 94.5, and 133.5 mmol/l, respectively) groups. Results showed no significant difference between the hypertonic and control groups in retinal endothelial cells and retinal ganglion cells at 24h, 48h, and 72h (7). Based on our published data, we did not repeat the experiment in the current study.

The limitation of this study is that this is a 2D co-culture *in vitro* system and only involved two cell types. Muller cells also play a significant role in maintaining the internal environment of RNVU, including supporting cell stability in RNVU, nourishing neurons, removing toxic substances, protecting neurons, and supporting normal cell membrane functions. There is growing evidence that chronic inflammation contributes to DR. Hyperglycemia also profoundly impacts microglial physiology, initiating a wide variety of microglial responses. Microglial cells act as modulators in the chronic inflammation process, in which microglia was activated at the transcriptional level, mediated through the nuclear factor kappa-light chain-enhancer of activated B cells and extracellular signal-regulated kinase signal pathways, resulting in the release of proinflammatory cytokine chemokines, caspases, and glutamate (23). Pericyte maintains the integrity of the blood-retinal barrier, increasing the expression of tight junction proteins by interactions with endothelial cells (24). Pericyte loss is the early pathological sign of DR (25). The interactions between microglial and endothelial cells, Müller cells, neurons, or pericytes have attracted more attention recently. Establishing a three-dimensional or involving three or more cell types in a cell culture system helps to understand the pathophysiological aspects of DR.

In summary, *in vitro* co-culture model for vascular and nerve coupling in DR can better stimulate the DR neurovascular coupling over a monoculture model. This study lays a technical and theoretical foundation for further research to understand DR. This culture mode can also help to find new biological biomarkers and molecular targets for DR.

## Data availability statement

The original contributions presented in the study are included in the article/supplementary materials, further inquiries can be directed to the corresponding author.

## Author contributions

QW performed the experiment and statistical analysis and drafted the manuscript. ZQ and WK instructed and joined in the experiments. LZ instructed and revised the manuscript. XZ drafted and revised the manuscript. All authors contributed to the article and approved the submitted version.
